# Cerebral sparganosis in a child with corpus callosum invasion: a case report

**DOI:** 10.1186/s12879-023-08322-9

**Published:** 2023-05-25

**Authors:** Xiwen Chen, Huiying Wu, Lianwei Lu, Ning Zhou, Zhen Chen, Xiaochun Zhang

**Affiliations:** Department of Radiology, Guangzhou Women and Children’s Medical Centre, Guangzhou, China

**Keywords:** Sparganosis, Parasitic diseases, Corpus callosum, Child, Magnetic resonance imaging, Migration mode

## Abstract

**Background:**

Invasion of the corpus callosum by sparganosis is rare in children. After invading the corpus callosum, sparganosis has various migration modes, which can break through the ependyma and enter the ventricles, thus causing secondary migratory brain injury.

**Case presentation:**

A girl aged 4 years and 7 months presented with left lower limb paralysis for more than 50 days. Blood examination showed that the proportion and absolute number of eosinophils in the peripheral blood were increased. Furthermore, enzyme-linked immunosorbent assay of serum and cerebrospinal fluid samples revealed positivity for IgG and IgM antibodies for sparganosis. Initial magnetic resonance imaging (MRI) revealed ring-like enhancements in the right frontoparietal cortex, subcortical white matter, and splenium of the corpus callosum. Within 2 months, a fourth follow-up MRI showed that the lesion had spread to the left parietal cortex, subcortical white matter, and deep white matter in the right occipital lobe and right ventricular choroid plexus, with left parietal leptomeningeal enhancement.

**Conclusion:**

Migratory movement is one of the characteristics of cerebral sparganosis. When sparganosis invades the corpus callosum, clinicians should be aware that it may then break through the ependyma and enter the lateral ventricles, leading to secondary migratory brain injury. Short-term follow-up MRI is necessary to evaluate the migration mode of sparganosis and dynamically guide treatment strategies.

## Background

Cerebral sparganosis accounts for approximately 25% of sparganosis cases [1]. The main route of infection is the digestive tract, such as from drinking water containing Daphnia or from eating raw or undercooked meat of frogs or snakes meat [2], [3]. Early diagnosis of cerebral sparganosis in children is difficult because of the low specificity of clinical manifestations, the limited expressive language abilities of children, and difficulties in obtaining an epidemiological history [2]. Clinical symptoms vary depending on the location and scope of the lesion; migration sign, tunnel-sign, worm-body signs, and ring-like enhancement are the magnetic resonance imaging(MRI) features of cerebral sparganosis that can aid in its early diagnosis [4–7]. MRI is also an important method for dynamically observing the migration mode of sparganosis which causes mechanical and chemical damage to brain tissue, and for evaluating the effects of treatment [4], [8]. Surgery is the optimal treatment for cerebral sparganosis [9], [10]; however, for patients who are unsuitable for surgery, high-dose quinolones can be used treat infection [11], [12].

Invasion of the corpus callosum by sparganosis is rare. Herein, we describe a case of sparganosis infection in a child with severe sequelae of invasion of the corpus callosum. Over a 2-month period, four brain MRIs were performed at our hospital to provide a comprehensive assessment of the the parasite’s trajectory. Our findings highlight the clinical importance of corpus callosum invasion for prognosis.

## Case presentation

**Fig. 1:**
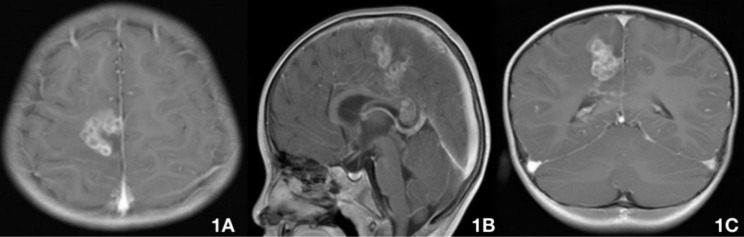
Brain MRI on July 23 (praziquantel 10 mg/kg/day for 3 days) showing multiple lesions in the right frontoparietal cortex, subcortical white matter, deep white matter and splenium of the corpus callosum (A–C). The ring-shaped enhancement lesions, 2.3–5.7 mm in diameter, are arranged in clusters

**Fig. 2:**
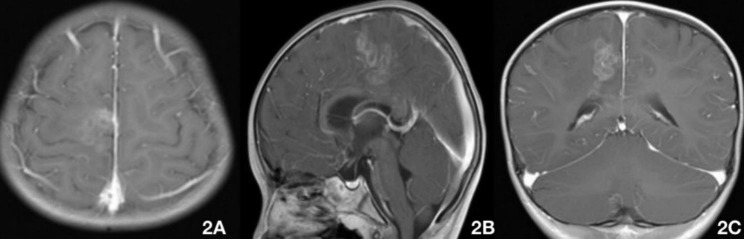
Brain MRI on August 5 (praziquantel 10 mg/kg/day for 3 days + 25 mg/kg/day for 13 days), showing lesions in the right frontoparietal cortex and splenium of the corpus callosum (A–C). The degree of enhancement was lower than that in the previous MRI (July 23)

**Fig. 3:**
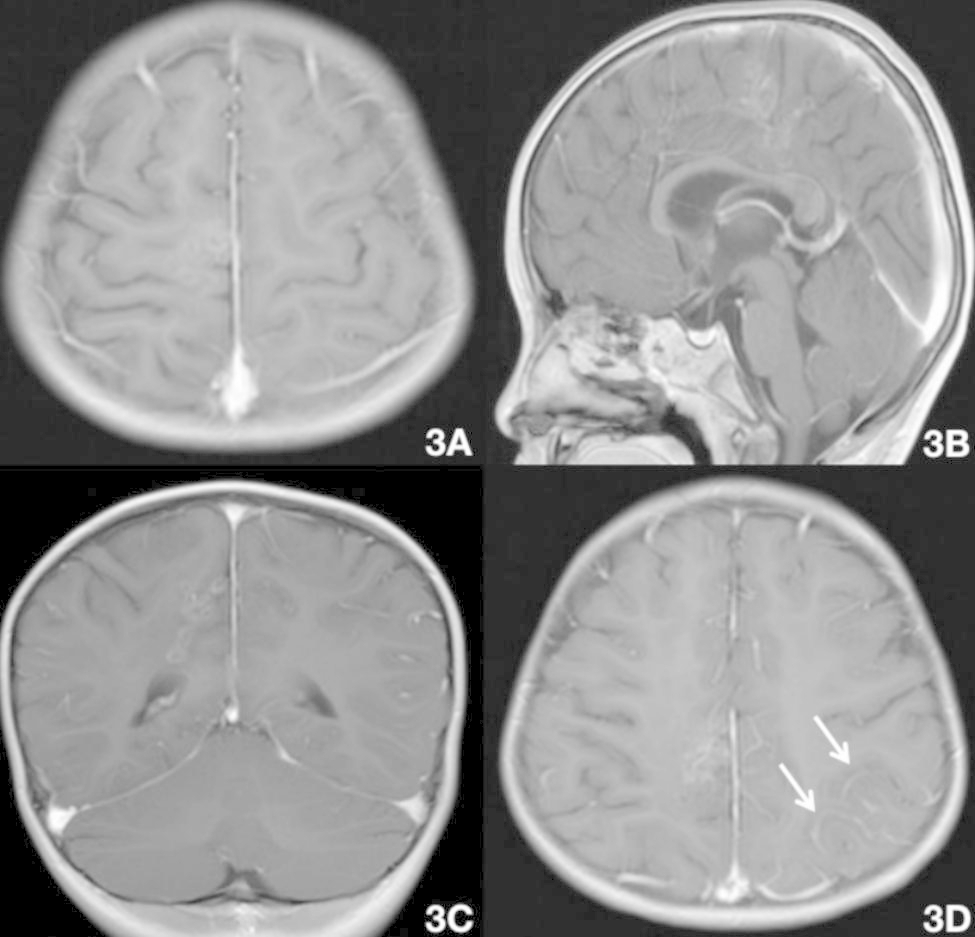
Brain MRI on August 17 (praziquantel 10 mg/kg/day for 3 days + 25 mg/kg/day for 25 days). The right frontoparietal lobe and the splenium of the corpus callosum were enhanced. The degree of enhancement in the foci (A–C) was lower than in the previous MRI (August 5), and pia mater enhancement was newly seen (D; white arrow, left top)

**Fig. 4:**
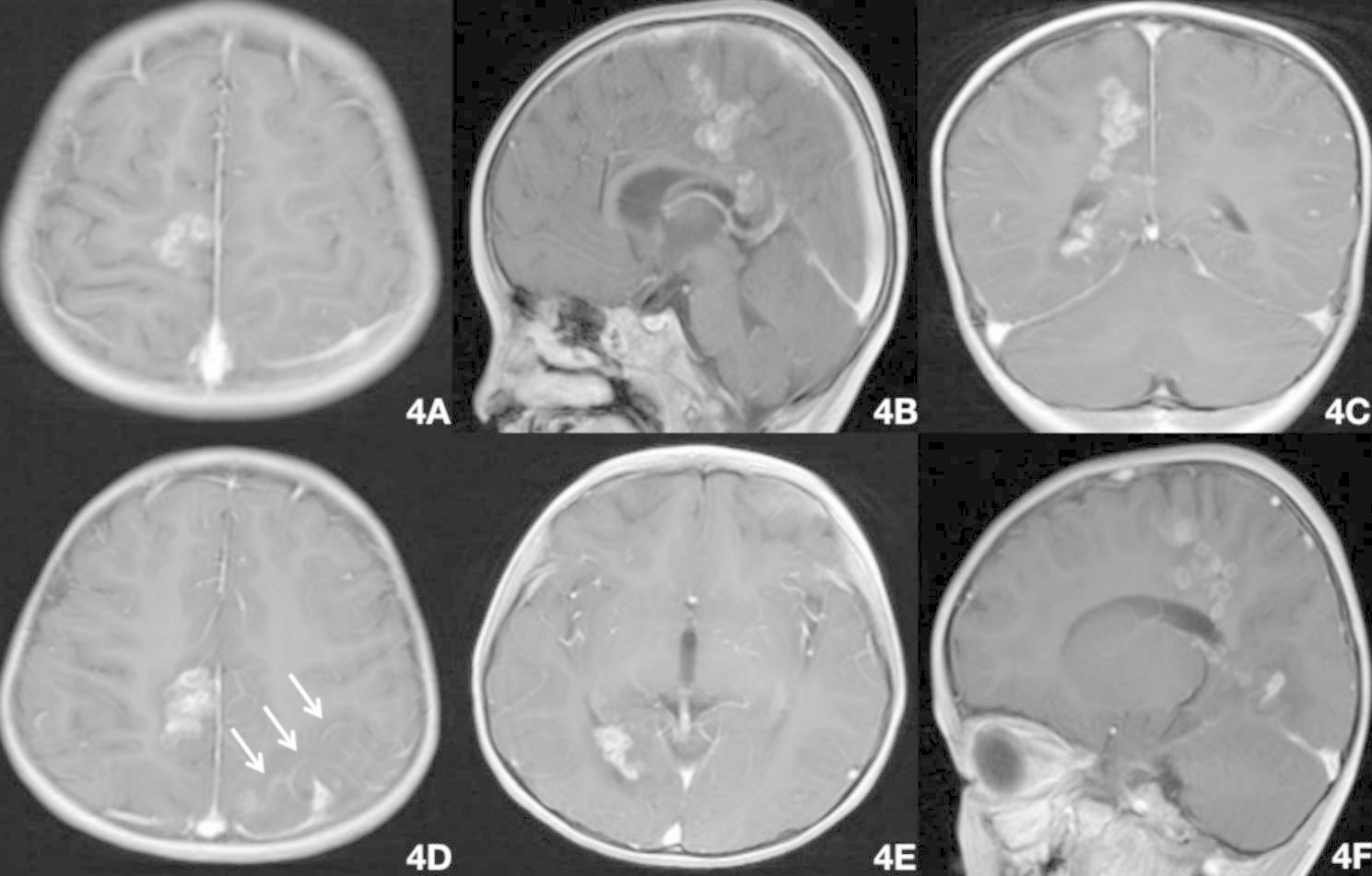
Brain MRI on September 8 (praziquantel 10 mg/kg/day for 3 days + 25 mg/kg/day for 26 days + 20 mg/kg/day for 7 days), showing lesions in the right frontoparietal lobe and splenium of the corpus callosum (A–C). The degree of enhancement was greater than in the previous scan (August 17), with multiple circular enhancements in the left parietal cortex and subcortical white matter (D), and in the deep right occipital lobe white matter and right ventricle choroid plexus (E,F). The area of pia mater enhancement was enlarged(D; white arrow, left top)

A girl aged 4 years and 7 months, who lived in a rural area of Guangxi Province(China), was admitted to the Neurology Department of Guangzhou Women and Children’s Medical Center on July 14, 2015, with left lower limb paralysis lasting more than 50 days. The girl had developed weakness in her left lower limb after falling more than 50 days previously, and occasionally had pain in her left knee when walking. On July 10, an enhanced MRI from another hospital showed multiple irregular ring-like enhancement lesions in the right frontoparietal lobe and the splenium of the corpus callosum, suggesting inflammatory lesions. Her clinical manifestations were increased muscle tension in the left lower extremity, muscle strength grade IV, left Babinski sign (+), and ankle joint spasm (+). Blood examination on July 15 showed that the percentage of eosinophils in her peripheral blood was 6% (normal reference value: 0.5–5%), and the absolute count was 0.84 × 10^9^/L (normal reference value: <0.6 × 10^9^/L). Enzyme-linked immunosorbent assay of cerebral parasites (*Schistosomes*, *Sparganosis*, *Taenia solium*, *Toxoplasm*, and *Paragonimia*) in the serum and cerebrospinal fluid showed IgG (+) and IgM (+) for sparganosis on July 16. On July 17, further enquiry by a clinician revealed that the child had eaten raw frog meat. Four brain MRI (Figs. 1, 2, 3 and 4) were performed at our hospital over an 8-weeks period, including three during hospitalization (July 23, August 5, and August 17, 2015). From July 21 to August 18, the patient received 10-25 mg/kg/day praziquantel as diagnostic treatment because expert consultation did not fully support a diagnosis of cerebral sparganosis. On August 19, the child’s symptoms improved and she was discharged according to the doctor’s advice (praziquantel 20 mg/kg/day for 7 days; oral prednisolone acetate 5 mg twice daily for 7 days followed by 5 mg once daily for 3 days). On September 8, a follow-up MRI (the fourth MRI) was performed in the outpatient clinic, and the doctor informed the patient’s guardians that surgical intervention may be required. In November 2015, the child underwent craniotomy at a local hospital, and a 6-cm-long sparganum in the right occipital lobe. At present, the child has weakness in both lower limbs because of brain tissue injury and is undergoing rehabilitation treatment, according to reports from her guardians. She has not yet returned to our hospital for physical examination or follow-up MRI.

## Discussion and conclusions

We searched the PubMed database using the terms “sparganosis”, “migration”, “brain” and “MRI”, and found just three articals that reported three cases of sparganosis with corpus callosum invasion by follow-up MRI (Table 1) [4], [5], [13]. In 2007, Kim et al. [4] provided the first MRI-based report of the trajectory of sparganosis invading the contralateral cerebral hemisphere from the right temporal-occipital lobe through the splenium of the corpus callosum. Feng et al. [5] summarized the movement trajectories of 22 cases of cerebral sparganosis into the following four types: 1) interlobar migration to areas of close proximity within the same lobe or to the adjacent lobe, usually limited to the same hemisphere ; 2) cerebellum–brainstem migration in which migration occurred from the cerebellum to the brainstem or vice versa ; 3) transventricular migration involving ipsilateral or contralateral migration through the ventricles ; and 4) transmidline migration in which the worm migrated through midline structures to the ipsilateral or contralateral hemisphere. In the present case, by comparing the fourth MRI with the first MRI at our hospital, we tracked the movement of sparganosis and divided its trajectory into four stages. Firstly, the sparganosis traveled from the right frontoparietal cortex to the splenium of the corpus callosum. Second, it entered the lateral ventricle through the splenium. Third, it invaded the choroid plexus of the right lateral ventricle(from dorsal to ventral). Finally it broke through the right lateral ventricle and invaded the deep white matter of the right occipital lobe. The movement process of sparganosis took just 2 months from the first MRI(in another hospital) to the last MRI(in our hospital), there were only 3 weeks between the last two MRI scans. The course of the disease thus progressed relatively rapidly. Furthermore, the present case report is the first to simultaneously show three types of cerebral sparganosis with distinct movement trajectories. Sparganosis invading the corpus callosum is likely more harmful than that invading the contralateral cerebral hemisphere. The cerebrospinal fluid system is extensive, providing numerous trajectories for sparganosis. In severe cases, the brain parenchyma can be invaded more than once(as in present case), and the spinal cord may even invaded [14], further increasing treatment difficulties.

**Table 1:** Summary of baseline and follow-up MRI data of patients with cerebral sparganosis in the corpus callosum from the available literature in PubMed

Age (yrs)/Sex/Ref	Baseline MRI		Follow-up MRI		Migration mode
	Location of active lesions	Location of degenerative lesions	Interval between the follow-up and baseline MRI (month)	Location of migrated lesions	
24/M/ [4]	Not available	Right temporo-occipital lobe	36 ^A^	Splenium of corpus callosum, left temporo-occipital lobe	transmidline migration
16/M/ [5]	Genu of corpus callosum and left frontal lobe	No	1, 3 ^A^	Left frontal lobe (inward)	transmidline migration
24/M/ [13]	White matter in right temporal lobe	Genu of corpus callosum, left parietal lobe, basal ganglia, frontal lobe, and right frontal lobe	2, 4 ^A^, 14	Gray matter in the same lobe	interlobar migration

^A^Time points at which the migrated lesions were observed

When we compared the first three MRI scans from our hospital, the level of enhancement of the lesions in the right frontoparietal lobe and splenium of the corpus callosum gradually decreased, indicating that the lesion had improved. However, the third MRI showed thickening and enhancement of the left parietal pia mater for the first time. This finding suggests that the old and new lesions overlapped temporally and that the sparganosis invaded the pia mater, which would permit the invasion of the lateral ventricle through the splenium of the corpus callosum. The last MRI confirmed this trajectory of the sparganosis. When comparing the fourth MRI with the third MRI, the lesions in the right frontoparietal lobe and splenium of the corpus callosum were again markedly enhanced, demonstrating sparganosis movement with inflammatory cell infiltration. These two MRI changes may be related to multiple factors, including the use of praziquantel. There is no unified standard for the non-surgical treatment of cerebral sparganosis, and the efficacy a conventional dose of praziquantel is unsatisfactory [2]. The pharmacokinetics of praziquantel are characterized by a peak plasma concentration 2 h after taking the drug, followed by a rapid concentration drop. During the first half-life, the drug enters the cerebrospinal fluid rapidly, but enters the worm body at a slow rate [11], [12]. Long-term high-dose praziquantel treatment (50 mg/kg/day for 10 days per month [11], or 70 mg/kg/day for 10 days every 3 months [12]) is effective for killing the parasites. In the present case, the child was treated with 10-25 mg/kg/day for 30 days during hospitalization and 20 mg/kg/day for 7 days after discharge. Thus, the dose of praziquantel was relatively low and the course of treatment was short. In addition, the worms may not have been completely eliminated by this treatment.

Cerebral sparganosis needs to be differentiated from other brain parasitic diseases (paragonimiasis, schistosomiasis, or cysticercosis), as well as inflammatory granulomas (brain tuberculosis or brain abscess) [15] and anaplastic pleomorphic xanthoastrocytoma [16] using imaging. Serological examination is helpful for distinguishing sparganosis from other parasitic diseases, and epidemiological history and laboratory examination are helpful for distinguishing sparganosis from inflammatory granuloma. Furthermore, magnetic resonance spectrum and perfusion-weighted imaging are helpful for discriminating between sparganosis and anaplastic pleomorphic xanthoastrocytoma.

In summary, sparganosis with corpus callosum invasion is a serious event that should attract the attention of radiologists and clinicians. Radiologists should make sure that it is mentioned in the imaging diagnosis report, inform clinician of the possible risks, and pay close attention to the development of the disease. Clinicians should shorten the interval between follow-up brain MRI according to the specific conditions of each patient, and organize multidisciplinary consultations in good time, and formulate treatment plans. When medical treatment is ineffective, clinicians should actively communicate with families that surgery is needed as early as possible to reduce the risk of long-term brain damage.

There are some limitations in the present study. Given the rare occurrence of sparganosis with corpus callosum invasion, the application of follow-up MRI to track the movement trajectory of parasites needs to be investigated in future studies. Although our patient received surgery and rehabilitation at a local hospital, we were unfortunately unable to obtain relevant medical records and follow-up MRI data; this was despite doing our best to communicate with the hospital, and with consent from the patient’s guardians.

Her surgery process, pathological results, and prognosis were therefore reported by her guardians.

## Data Availability

The datasets used and/or analyzed during the current study are available from the corresponding author on reasonable request.

## Abbreviations

MRI: Magnetic resonance imaging
